# A New Hybrid Sensitive PANI/SWCNT/Ferrocene-Based Layer for a Wearable CO Sensor

**DOI:** 10.3390/s21051801

**Published:** 2021-03-05

**Authors:** Mihaela Savin, Carmen-Marinela Mihailescu, Viorel Avramescu, Silviu Dinulescu, Bogdan Firtat, Gabriel Craciun, Costin Brasoveanu, Cristina Pachiu, Cosmin Romanitan, Andreea-Bianca Serban, Alina Catrinel Ion, Carmen Moldovan

**Affiliations:** 1 National Institute for Research and Development in Microtechnologies, Erou Iancu Nicolae Street 126A, 077190 Bucharest, Romania; mihaela.savin@imt.ro (M.S.); viorel.avramescu@imt.ro (V.A.); silviu.dinulescu@imt.ro (S.D.); bogdan.firtat@imt.ro (B.F.); gabriel.craciun@imt.ro (G.C.); costin.brasoveanu@imt.ro (C.B.); cristina.pachiu@imt.ro (C.P.); cosmin.romanitan@imt.ro (C.R.); 2 Department of Applied Chemistry and Materials Science, University Politehnica of Bucharest, 1-7 Polizu, 011061 Bucharest, Romania; alina.ion@upb.ro; 3 Doctoral School in Engineering and Applications of Lasers and Accelerators, University Politehnica of Bucharest, 060042 Bucharest, Romania; andreea.serban@eli-np.ro; 4 Extreme Light Infrastructure-Nuclear Physics (ELI-NP), ‘Horia Hulubei’ National R&D Institute for Physics and Nuclear Engineering (IFIN-HH), 30 Reactorului Street, 077125 Măgurele, Romania

**Keywords:** wearable sensor, polyaniline, single-walled carbon nanotubes, polymeric nanocomposite material, gold interdigitated sensor, ferrocene, monoxide, electropolimerization

## Abstract

Developing a sensing layer with high electroactive properties is an important aspect for proper functionality of a wearable sensor. The polymeric nanocomposite material obtained by a simple electropolymerization on gold interdigitated electrodes (IDEs) can be optimized to have suitable conductive properties to be used with direct current (DC) measurements. A new layer based on polyaniline:poly(4-styrenesulfonate) (PANI:PSS)/single-walled carbon nanotubes (SWCNT)/ferrocene (Fc) was electrosynthesized and deposed on interdigital transducers (IDT) and was characterized in detail using electrochemical impedance spectroscopy (EIS), cyclic voltammetry (CV), scanning electron microscopy (SEM), Raman spectroscopy, X-ray photoemission spectroscopy (XPS), and X-ray diffraction (XRD). The sensor characteristics of the material towards carbon monoxide (CO) in the concentration range of 10–300 ppm were examined, showing a minimal relative humidity interference of only 1% and an increase of sensitivity with the increase of CO concentration. Humidity interference could be controlled by the number of CV cycles when a compact layer was formed and the addition of Fc played an important role in the decrease of humidity. The results for CO detection can be substantially improved by optimizing the number of deposition cycles and enhancing the Fc concentration. The material was developed for selective detection of CO in real environmental conditions and shows good potential for use in a wearable sensor.

## 1. Introduction

The synthesis of solutions based on conductive polymeric nanocomposite materials (PNCMs), as well as exploring their electrical properties, continues to be of great importance for a wide range of applications, such as sensors [1,2] and actuators, biosensors [3], micro/nanoelectronic devices, photovoltaic devices, supercapacitors [4,5], and light emitting diodes. PNCMs can be easily obtained from conductive polymers such as polyaniline (PANI), polypyrrole (PPy), polythiophene (PTh), and polyethylene (3,4-dioxythiophene) (PEDOT), which manage to increase their solubility in water (improve dispersion) by combining with other hydrophilic polymers such as poly (4-styrenesulfonate) (PSS), polyacrylic acid (PAA), poly(vinylpyrrolidone) (PVP), and polyethylene glycol (PEG). This results in composite solutions, such as [6,7], PANI:PEG [8], PANI:PVP [9], PEDOT:PSS [10,11], and PPy:PSS [12], with high conductivity and good optical properties. Conductive polymers and composite solutions can be combined with other components, such as carbon nanoparticles, carbon nanotubes, graphene and graphite nanofibers, in order to obtain PNCMs, such as PEDOT:PSS/graphene [13], PANI:PSS/graphene [14], PANI:PSS/multi-walled carbon nanotubes (MWCNTs) [15], and PEDOT:PSS/single-walled carbon nanotubes (SWCNTs). The challenge to obtain a good dispersibility and stability of the carbon-based components in the solution remains. The insertion of MWCNTs in the PANI:PSS composite, even in small amounts, generates an increase in the electrical properties of PANI, as MWCNTs ensure charge transfer efficiency [15,16]. In another instance, the combination of lower mass proportion carbon nanotubes (SWCNTs), which have one-dimensional structures and nanometric dimensions, with conductive polymers will determine an improvement in the electrical and mechanical properties of the obtained conductive solutions [17,18]. To solve the solubility problem, therefore improving the electrical properties of the nanocomposite polymeric solutions (given the fact that SWCNTs are hydrophobic and therefore insoluble in most solvents), would require finding suitable dispersion methods. These methods may include SWCNT surface functionalization, the addition of surfactants [19], the ultrasonication or the association of the other polymers, biomolecules, organic acids or organic sulfonates [20,21].

The influence of humidity remains one of the major challenges of gaseous sensors, especially for carbon monoxide (CO) detection. It is known that PANI responds well to humidity by decreasing the resistance [22,23], but many previous studies of gas detection do not discuss the influence of humidity. This parameter is of great importance since the sensitivity of the gaseous sensor is highly dependent on the resistance of this sensor in the air. Many authors use the sensitivity value, S (%), which is defined as the relative change in the electrical resistance using the following equation [24]:

(1) $$S\left(\%\right)=\frac{\left|R_{g}-R_{a}\right|\;}{R_{a}}*100\;$$

where $R_{g}$ is the resistance value in the presence of gas and *R_a_* is the resistance value in air. PANI has good chemical stability, it is easily obtained as a homogenous layer, and due to its two different oxidation states, it is suitable for gas sensing applications [25,26]. Generally, PANI has a stratified structure, but with the incorporation of carbon nanotubes, an interconnected mesoporous network structure is formed.

This conductive nature of the PANI/carbon nanotube composite can increase the rate of electric charge carriers. As a result, the number of active sites will grow, which effectively increases the intra- and inter-chain charge mobility in the presence of the donor electrons or the acceptor gases. Therefore, the use of a combination such as PANI with MWCNT, SWCNT or graphene has provoked the interest of many researchers. However, only a few discuss the interference of humidity. Zhang et al. showed that the humidity sensitivity of the PNCMs may be lowered or even cancelled out by precisely functionalizing SWCNT networks with camphor sulfonic-acid-doped polyaniline. If there is a proportion between functionalized and unfunctionalized SWCNT, the humidity influence gets cancelled out effectively. The opposite electrical responses of the two materials (PANIs decreased resistance and SWCNTs increased resistance) are determined by the presence of water molecules in the air [27].

The increased sensitivity of conductive materials used to detect CO has also been the subject of several studies to date. For instance, Shimizu et al. have increased the CO sensitivity by introducing perovskite-type oxides on gold interdigitated electrodes on an Al_2_O_3_ substrate. These sensors showed the disadvantage of optimum operating at temperatures of 400 °C [28]. Thus, the use of a co-dopant such as ferrocene (Fc) or ferrocenyl derivatives has been shown to be a method that could improve the response to monoxides at room temperature [29]. Polypyrrole functionalized with the ferrocenyl derivate was used by other authors for detecting CO with high sensitivity (<100 ppm). Other findings have shown an enhanced electrochemical property when a noncovalent nanohybrid of Fc was used in combination with SWCNT [30,31]. Therefore, a combination of a conductive polymer with SWCNT covalent/noncovalent bonding with Fc/Fc derivates may have potential for CO detection with a negligible humidity interference.

The deposition method presents another challenge in the formation of a sensor with NCPMs. If one selects the method of easy controlling of the parameters, one could obtain a reproducible sensor with a good sensitivity. The synthesis and deposition of some NPCMs on the substrates can be obtained by different methods, such as spin coating [32], dip coating [33], thermal evaporation [34], Langmuir-Blodgett [35], inkjet printing [36], and electrochemical methods [37].

The electrochemical method is well known and often used for the synthesis of conductive polymers on the solid substrates, since the formation and properties of the substrate (topography and thickness) can be controlled not only by the conditions under which the electrosynthesis takes place (voltage control, potential electrodes, cycle numbers, and deposition time), but also for low cost and simplicity [38,39,40,41]. If an appropriate nanocomposite polymeric solution with high homogeneity for electropolymerization (EP) is used, the process can easily be optimized in order to obtain good surface coverage, even if CNT materials are used, knowing that they have low solubility.

Some of the most important microsensors for environmental monitoring devices are those that detect polluting and explosive gases. One of the key uses for microsystems technologies in environmental monitoring is for quantifying human exposure. Measuring the quality of the air or the various polluting or dangerous substances in the air (gaseous, volatile compounds, etc.) allows extremely important data regarding exposure factors to be obtained, such as the level of exposure, the exact location, and the time when they appear. All this information, correlated with medical data, significantly contributes to the overall picture of the cumulative effect of exposure to environmental factors. Wearable monitoring systems may provide a solution in promoting wellness management and rehabilitation outside clinical environments, when used as smart sensor nodes integrated into a portable device with a reasonably small form factor, that does not hinder or limit the individual’s activity [42].

Development of a sensing layer with high electroactive proprieties is important for proper functionality of wearable sensors [43]. The NCPMs and the CPs are used in the wearable application since they have good electromechanical stability and electroactive properties [44].

The aim of the present work is to use EP to obtain a sensing composite film base of PANI:PSS/SWCNT/Fc with potential applicability in CO detection, which is miniaturized and wearable. The number of cyclic voltammetry (CV) cycles, together with the rate of PANI versus SWCNT, were found to be the most important parameters to eliminate cross sensitivity with humidity. This can be obtained by varying the times of deposition or the number of CV cycles. The electrochemical characterization, along with the scanning electron microscopy (SEM), Raman, XPS and XRD investigations, were performed after each stage of the film preparation. The electroactivity and the sensitivity were increased after the addition of the Fc on the sensitive layer. The sensors were integrated in an Internet of Things (IOT) wearable self-monitoring system contained in a readout module connected to the sensor in the test chamber. Data logging was established through a smartphone wireless connection.

## 2. Materials and Methods

### 2.1. Materials

Aniline (anhydrous, >99.5%), poly(4-styrenesulfonic acid) sodium salt (PSS) with mol wt. (M_w_) ~70,000, carboxylic acid functionalized SWCNTs (>90% carbon basics, diameters from 4 to 5 nm and lengths from 0.5 to 4 μm), 1,1 ferrocene dimethyl, sulfuric acid (95–98%), nitric acid 70% (HNO_3_), ethyl alcohol pure, potassium chloride (KCl), phosphate buffer saline (PBS, pH 7.0), K_3_[Fe(CN)_6_] and K_4_[Fe(CN)_6_] were purchased from Sigma-Aldrich. All solutions were prepared using Milli-Q deionized water.

### 2.2. Methods

#### 2.2.1. Solutions Preparation

- Solution S1: 0.3 M aniline monomer was dissolved in 0.5 M H_2_SO_4_ in 24 mL of deionized water.
- Solution S2: PANI:PSS/SWCNT was prepared as follows: 3.2% PSS was dissolved in 24 mL of deionized water and ultrasonicated. Over the PSS dissolved solution, 0.01% SWCNT and 0.5 M/L H_2_SO_4_ were added and the ultrasonication was continued for 2 h after which it was magnetically stirred by heating at 50 °C for another 2 h. After the SWCNT was dissolved in the solution, an extra 0.3 M/L of monomer aniline was added.
- Solution S3: The Fc solution preparation was performed as follows: 4 mM/L Fc was added to 3 mL of absolute ethanol, 600 µL of HNO_3_, and 1.5 M of H_2_SO_4._
- Solution S4: PANI:PSS/SWCNTs/Fc. The S4 solution used in the electrosynthesis has resulted from the mixing of S2 and S3 solutions in a ratio of 3:1 (*v*/*v*).

#### 2.2.2. Electrochemical Experiments

All electrochemical experiments were performed using VoltaLabPGZ100. All deposition experiments were carried out using conventional three electrode systems. The IDEs were used as the working electrode, the counter electrode was Pt, and the reference electrode was Ag/AgCl (3 mKCl). The electrochemical impedance spectroscopy (EIS) experiments were performed at an open circuit potential in the frequency range of 100 kHz and 0.1 Hz with a 10 mV amplitude in a 0.5 mM ferri/ferrocyanide redox probe (PBS 7.4, 0.1 M was the supporting electrolyte solution).

- EP conditions to obtain PANI layers: The sensors were immersed in the electrolyte solution, S1 solution (ANI in H_2_SO_4_), and the CV was performed by linear scanning at −200 and 900 mV with a scanning speed of 50 mV/s for 40 cycles.
- EP conditions for PANI:PSS/SWCNTs layers: The sensors were immersed in a composite solution, S2 solution (PANI:PSS/SWCNTs), and the CV was performed by linear scanning at −200 and 900 mV with a scanning speed of 50 mV/s for 40 cycles.
- EP conditions for PANI:PSS/SWCNT/Fc layers: The sensors were immersed in a S4 solution, PANI:PSS:SWCNT:Fc, and the CV was performed by linear scanning at −200 and 900 mV with a scanning speed of 50 mV/s for 40 cycles.

#### 2.2.3. Raman Spectroscopy

The Raman spectra were collected at room temperature with a WITec Raman spectrometer (Alpha-SNOM 300 S, WITec GmbH, Ulm, Germany) using 532 nm as an excitation. The 532 nm diode-pumped solid-state laser has a maximum power of 145 mW. The incident laser beam with a spot-size of about 1.0 µm was focused onto the sample with a 6 mm working distance objective attached to a Thorlabs MY100X-806 microscope. The Raman spectra were measured with an exposure time of 20 s accumulation and the scattered light was collected by the same objective in back-scattering geometry with 600 grooves/mm grating. The calibration of the Raman systems was carried out using the 520 cm^−1^ Raman line of a silicon wafer. The spectrometer scanning data collection and processing were carried out by a dedicated computer using the WITec Project Five software.

#### 2.2.4. X-ray Photoemission Spectroscopy (XPS)

The XPS spectra were recorded on a Sigma Surface Science photoelectron spectrometer equipped with a 160-mm hemispherical energy analyzer with a 1D detector (ASPECT) and using an Al Kα X-ray source at 13 kV at a power of 200 W. The analysis area was 1.3 × 1.3 mm^2^. The pressure in the analysis chamber was kept below 1 × 10^−9^ mbar. Wide scan survey spectra were collected from −5 to 1200 eV (binding energy), using constant pass energy of 289 eV. The high-resolution spectra of O1s, C1s, and N score levels were recorded using a pass energy of 50 eV. Since the Fe 2p signal was very weak, the spectra were collected for a 10-times longer acquisition time than the other high-resolution spectra. The CasaXPS software was used for analysis. All spectra were calibrated by the adventitious carbon C1s main peak to be at 284.8 eV. All spectra were fitted using a Shirley type background and a Lorentzian Asymmetric peak-shape (LA(1.53,243)). Prior to the XPS measurements, the samples were infrared heated to ~100 °C for 5 min.

#### 2.2.5. X-ray Diffraction (XRD)

The X-ray patterns were recorded using a 9 kW Rigaku SmartLab diffractometer with Cu rotating anode in grazing incidence (GI), with a small incidence angle of 0.5°, while the detector angles was varied between 5 and 80°. The experimental GIXRD patterns were further analyzed in the PDXL software developed by Rigaku and the peak indexing was achieved using the International Center of Diffraction Data (ICDD) database. To assure a good resolution in the experimental patterns, a narrow soller slit of 0.5° was used at the detector.

#### 2.2.6. SEM Spectroscopy

The SEM micrographs were obtained using a field emission gun scanning electron microscope (FEI Nova™ NanoSEM 630, Hillsboro, OR, USA). This produces enlarged images of a variety of specimens, achieving magnifications of over 500,000×, providing ultra-high resolution imaging in a digital format. This important and widely used analytical tool provides exceptional depth of field, minimal specimen preparation, and the ability to combine the technique with X-ray microanalysis.

#### 2.2.7. Sensor Fabrication

The sensor was fabricated on a silicon substrate using standard photolithographic fabrication techniques. An Si wafer underwent thermal treatment to grow the SiO_2_ on top by cleaning it in H_2_SO_4_ at 50 °C and was treated at 200 °C before spinning the photoresist layer (1 µm) and UV exposing the electrode mask (M1), as shown in Figure S1a. After the photoresist development, the next step was the metal deposition of Ti/Au (15/200 nm), followed by lift-off. Plasma-enhanced chemical vapor deposition (PECVD) Si_3_N_4_ was deposited (300 nm) and patterned (Mask 2), leaving open the interdigital transducers (IDTs) and pads areas. The IDTs consisted of 100 pairs of IDEs, each digit having 10 µm width and 10 µm gap between the digits. The size of the IDE area on the sensor was 0.4 × 0.4 mm^2^. Figure S1b shows the image of the IDTs used for fabrication. The digit pairs were routed to two pads on one side of the chip for rapid interconnection with electronic readouts. The electrode pads were designed to enable direct connection into a standard connector.

#### 2.2.8. Gas Sensing Measurements

Before testing, the sensor was mounted in a chamber and the testing gas was flown into the chamber. The testing gas was prepared by mixing purified nitrogen with different ratios of CO (nitrogen was the carrier for CO) with a certificated concentration of 1000 ppm. The gas flow rate was manipulated by a computer controller using a multi-channel mass flow controller. The gas sensor was exposed at a concentration of CO for 300 s and then the sensor was recovered with exposure to nitrogen for another 300 s. The humidity tests took place at room temperature, ~20 °C, and under N_2_ flow. The calibration of the sensor for CO was made under N_2_ flow and under controlled conditions using the LabView based software. The resistances were continuously monitored by measuring the current. Figure S2 shows the installation for CO detection.

#### 2.2.9. Wearable Electronic Readout Module

**Figure 1:**
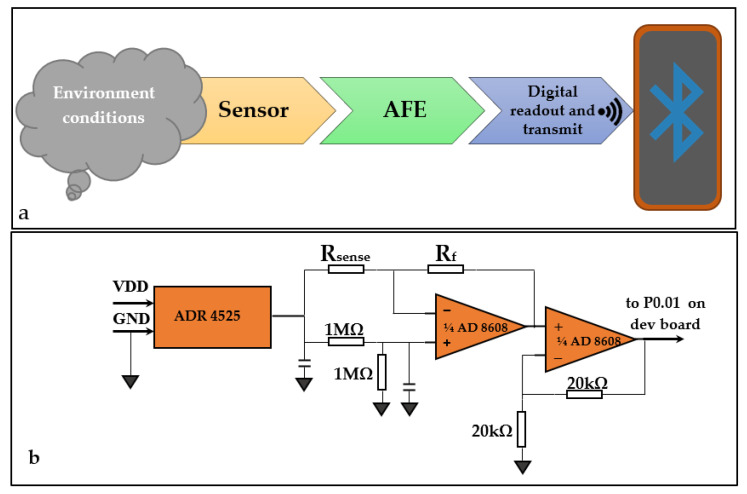
(**a**) Block diagram for the electronic module of the wearable device; (**b**) schematic of the analog front end (AFE).

In order to get data from the sensors, an electronic module for interfacing the sensor was developed. The sensor’s resistance value was converted to an analogue voltage level and then to digital, and was further sent to a smartphone via a wireless connection. The electronic readout module for the wearable device is given in a general block diagram, as shown in Figure 1a.

Figure 1b shows the schematic for the analogue front end (AFE) block. A bridge circuit along with a precision voltage reference was used in the AFE to effectively measure a voltage output proportional to the sensor’s electrical resistance, which in turn was related to the CO concentration. This output voltage was read by an analogue to digital converter (ADC) in the microcontroller and data were forwarded to a connected smartphone via a Bluetooth wireless connection.

The relation between the output voltage and the sensor’s resistance is given in the Formula (2) below:

(2) $$V_{out}=2.5\left(1-\frac{R_{f}}{R_{sense}}\right)$$

**Figure 2:**
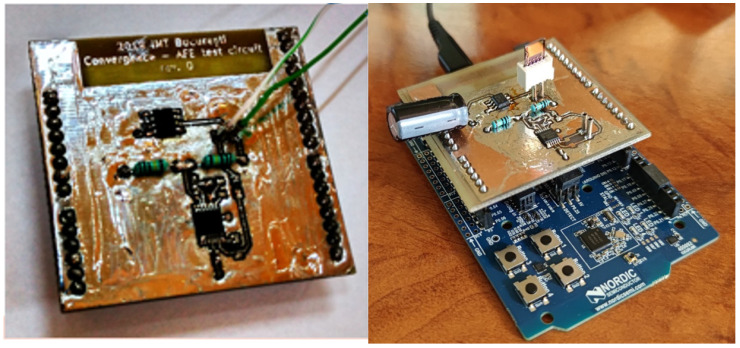
(**a**) Fabricated AFE circuit for interfacing the sensor to the microcontroller development board; (**b**) electronic readout test assembly.

The AFE block has been implemented as a shield for the Nordic Semiconductor nRF51 development board, as shown in Figure 2a. Measured data are sent to a smartphone application via the Bluetooth connection for data logging. The sensor is mounted in a connector onboard for easy swapping during experimenting, the whole setup being shown in Figure 2b.

The overall component selection for the electronics was made so that everything can be integrated in a later step into a single board solution, with only the strictly necessary functional features, resulting in a small form-factor solution for a frictionless user experience. Considerations were made regarding balancing low power consumption with the requirement to obtain accurate and reliable data.

## 3. Results and Discussion

### 3.1. Process and Material Analysis

#### 3.1.1. Visual Characterization of the S2 Solution

Supplementary Figure S3a shows the solution with aniline in sulfuric acid and SWCNT made without PSS. Figure S3b shows the solution with aniline in sulfuric acid and SWCNT after the addition of 3.2% PSS, respectively. A clear improvement in the solubility of the solution can be observed due to the introduction of the PSS polymer compared to the solution of aniline and carbon nanotubes in which this polymer was not introduced. These formulations further help obtain a homogenous deposition and therefore lead to reproductible electrical results of the film depositions.

#### 3.1.2. Optimization of EP Process on IDEs

**Figure 3:**
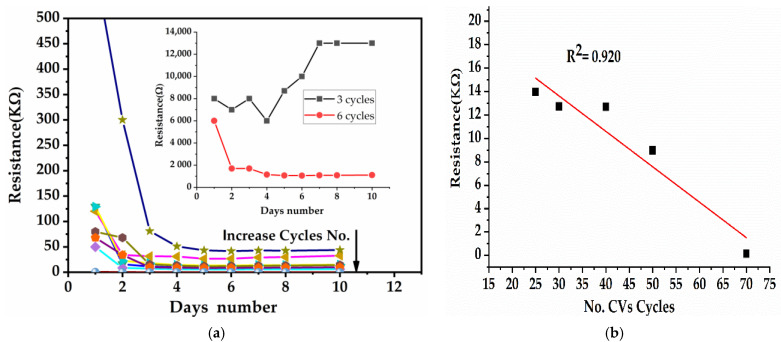
(**a**) Values of resistance during 10 days; (**b**) liniarity range of resistance versus cyclic voltammetry (CV) cycles number.

The optimization of the CV cycles was one of the important parameters for controlling the sensor performance. The resistance stability time for freshly deposed film was recorded during 10 days at room temperature and in dust-proof conditions, in order to test the long-term stability of initial resistance. The inset of Figure 3a clearly shows that at the beginning, the resistance obtained from a small number of cycles (three and six) deposed onto the IDEs had higher values at the MΩ level and were most unstable in the first 4 days. All resistance values stabilized after 7 days of storage and decreased to the KΩ level only if the number of EP cycles onto the IDTs increased from eight or more. These findings can be explained by insufficiently conductive composite materials deposed on the IDEs at the first EP cycle.

In order to achieve good reading sensitivity, the sensors on which the composite layer was deposited had to have a resistance range greater than 1 kΩ. Thus, it was necessary to optimize the SWCNT concentration, keeping the mass of aniline constant, so that the resistance after EP fell within resistances of over 1 kΩ. The conductive surfaces <100 Ω cannot be read with our systems, as this is the lower resistance limit at which the signal can be recorded without very high noise. The sensors were obtained by gradually applying CV cycles until the conductivity reached the Ω level.

We tried using the optimized concentration of SWCNT to find a range of resistances that was optimal to measure the CO. Thus, for 0.01% of SWCNT and 1.33 mM of Fc concentration, the resistance of the IDEs after 7 days became almost linear with the number of cycles. Figure 3b shows the linearity range between 70 and 20 cycles, a linearity coefficient R^2^ = 0.920, and resistance values between 0.142 and 14 kΩ. For an appropriate measurement with our systems, the IDEs with resistance values between 2 and 14 kΩ were chosen, to correspond with a number of 50 to 20 CV cycles.

Supplementary Figure S4 presents the sensors after successive EP deposition cycles: Six, eight, 15, 25, 40, and 70. The changes of colours of the IDEs area can be noticed. With the increase in the number of EP deposition cycles, the colour changed from brown (at lower numbers) to green (PANI emeraldine salt formed at 40 cycles), and eventually, to dark green (when the EP deposition reached around 70 cycles).

#### 3.1.3. Morphological and Structural Characterizations

- SEM characterization

**Figure 4:**
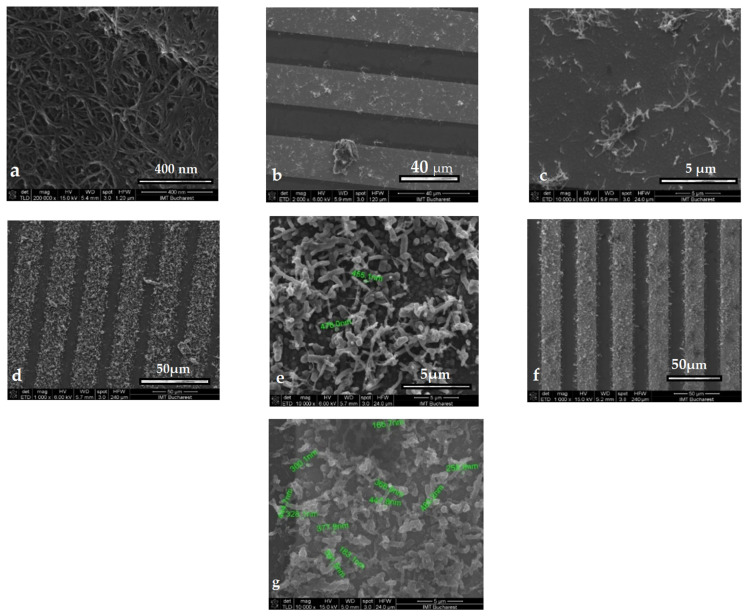
SEM images for (**a**) single-walled carbon nanotubes (SWCNTs) on interdigitated electrodes (IDEs); (**b**) and (**c**) 15th cycle electropolymerization (EP) of polyaniline:poly(4-styrenesulfonate) (PANI:PSS)/SWCNTs on IDEs; (**d**) 40th cycle EP of PANI:PSS/SWCNTs on IDEs; (**e**–**g**) 40th cycle EP of PANI:PSS/SWCNTs/ferrocene (Fc) on IDEs.

Figure 4a shows a morphology such as curly hollow ropes when the SWCNTs were analyzed by SEM. The PANI:PSS/SWCNT film on the IDEs were also analyzed by SEM at six, eight, 15, 25, and 40 cycles. After 20 cycles, the films became more homogenous than at lower cycles and the interaction between SWCNT and PANI produced a PANI coating on each CNT or CNT wisp. The observation was also reported by other study groups.

It is possible that the amount of PANI molecules covering the SWCNTs was dependent on the exposed area of nanotubes and on their morphological characteristics. Therefore, as shown in Figure 4b,c, after 15 CV cycles, the PANI:PSS/SWCNT composites did not form a compact layer, since the amount of SWCNT molecules were low and PANI only covered a few hollow ropes.

After the increase in the number of CV cycles (at 40), the layer became more compact and homogenous, as shown in Figure 4d,e. PANI appeared as some end-chopped fibrils. The diameter of these fibrils increased with the number of CV cycles. From an electrical point of view, a more compact film deposed on the IDEs led to a decrease in resistance values, but for our measurements, the optimal compact film with homogenous deposition and with resistance at the KΩ level was stabilized at 40 CV cycles. If the number of CV cycles exceeded 40, the resistance values decrease to Ω values.

Subsequent to adding the Fc in layers, the morphology (as presented in Figure 4f,g), showed a stronger interaction between composite components, that led to fewer extensions of the molecules between the IDEs compared to the layer presented previously in Figure 4d.

#### 3.1.4. Raman Characterization

**Figure 5:**
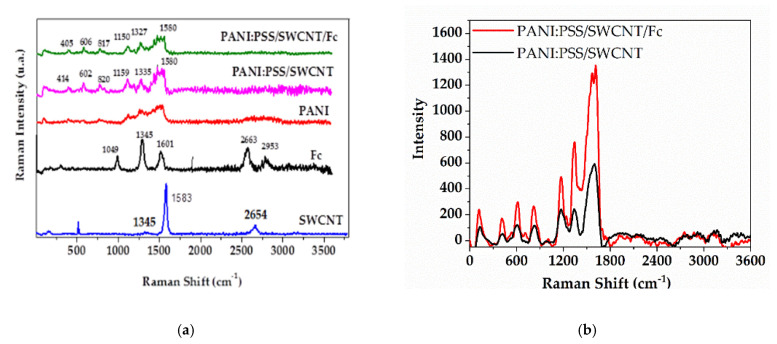
Raman characterization: (**a**) Raman spectra of SWCNT, Fc, PANI, PANI:PSS/SWCNT and PANI:PSS/SWCNT/Fc; (**b**) intensity bands of PANI:PSS/SWCNT compared to PANI:PSS/SWCNT/Fc.

As Figure 5a shows, all the samples had a disorder band (D band) at ~1345 cm^−1^ and a vibration of the sp^2^ carbon lattice (G band) at ~1580 cm^−1^. The D and G bands revealed that the structural defects on PANI, PSS, SWCNT, and Fc were intercalated. The adding of Fc did not provide an obvious signal on the PANI:PSS/SWCNT/Fc spectra, meaning that the Fc was well dispersed over the carbon materials. However, it can be observed that the Fc addition showed an increase in band intensity, as presented in Figure 5b, which is possible due to the noncovalent interaction of Fc molecules with the PANI:PSS/SWCNT composite film. H. Kuzmany et al. mentioned the same behavior after adding the Fc on SWCNT [45].

#### 3.1.5. XPS and XRD Characterizations

The chemical composition of the surface and the oxidation states of the elements in the samples were studied using photoelectron X-ray spectroscopy (XPS). As expected, the XPS survey spectra of the samples indicated the presence of species with C1s, O1s, N1s, and Fe 2p in the PANI:PSS/SWCNT/Fc layer and were presented in Figure S5 [46,47].

**Figure 6:**
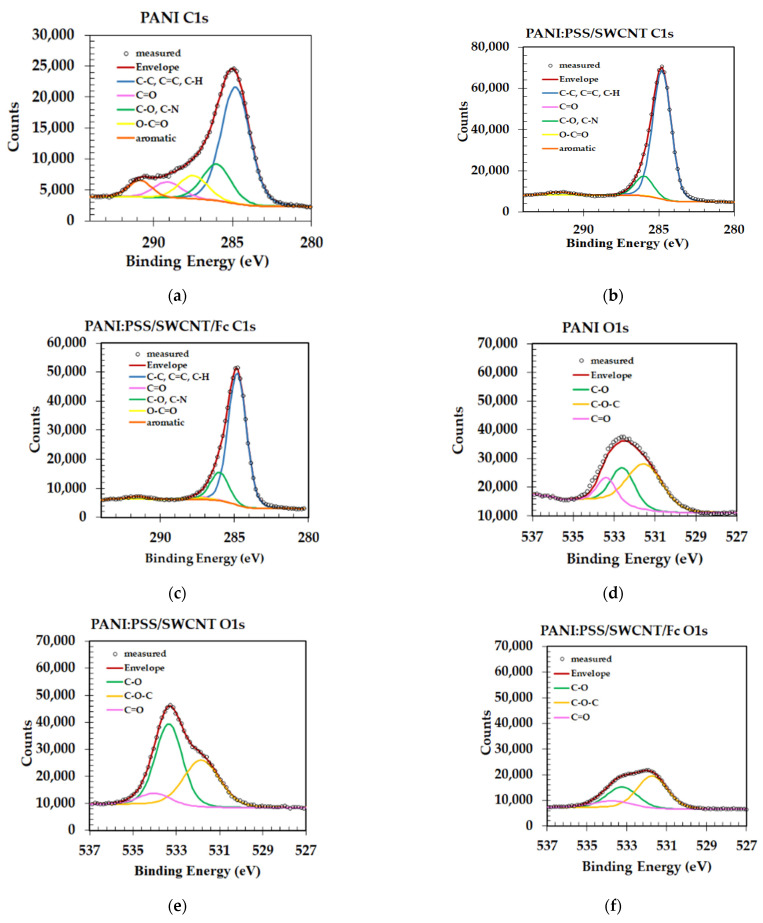

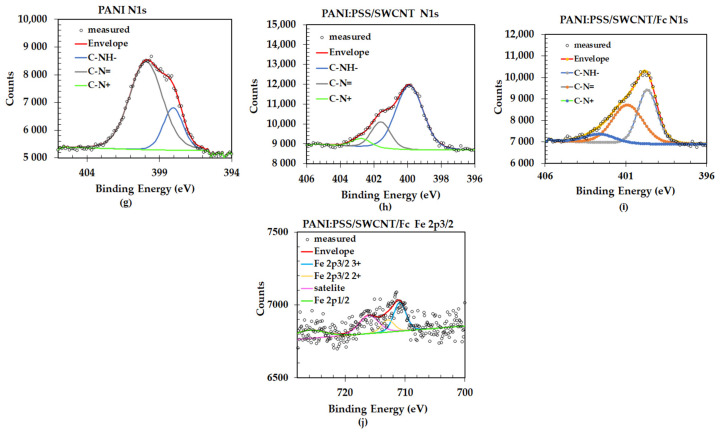
Spectra deconvolution of electrons from 1s orbital carbon C1s for (**a**) PANI; (**b**) PANI:PSS/SWCNT; (**c**) PANI:PSS/SWCNT/Fc; spectra deconvolution of of electrons from 1s orbital of oxygen, O1s for (**d**) PANI; (**e**) PANI:PSS/SWCNT; (**f**) PANI:PSS/SWCNT/Fc; spectra deconvolution of electrons from 1s orbital of nitrogen, N1s for (**g**) PANI; (**h**) PANI:PSS/SWCNT; (**i**) PANI:PSS/SWCNT/Fc; spectra deconvolution of of electrons from 2p orbital of iron, Fe 2p for (**j**) PANI:PSS/SWCNT/Fc.

Following high resolution scans in the 700–725 eV region, Fe 2p components in the PANI:PSS/SWCNT/Fc layer were observed. If it follows the C1s component after deconvolution, it can be seen in Figure 6a–c and Table 1 that the aromatic component in the PANI spectrum almost disappeared in the case of the PANI/SWCNT/Fc layer, probably due to the incorporation of ferrocene in the surface. Moreover, for this layer the C=C bonds were hidden in C-C and C-H, but were very difficult to separate. A possible explanation for the increase in the concentration of C-C, C=C, and C-H bonds is the incorporation of ferrocene that contains these bonds, since it has tw double cycles of cyclopentadienyl.

The peak deconvolution of O1s core-level spectra of PANI, PANI:PSS/SWCNT, and PANI:PSS/SWCNT/Fc samples revealed that three components were attributed to the oxygen in C=O, C-O, and C-O-C bonds. The C=O was highly visible in PANI comparative with PANI:PSS/SWCNT and PANI:PSS/SWCNT/Fc. The XPS spectra of O1s components are presented in Figure 6d–f and the distribution of components are presented in Table 2.

Figure 6g–i shows the spectra deconvolution of N1s with three components: C-N+, C-N=, and C-NH-. It was observed that in the PANI layers, the C-N^+^ did not exist, possibly due to the formation of the conducting polymer PANI as an emeraldine salt polaron/bipolaron form. The distribution of N1s component is presented in Table 3.

The peak deconvolution of Fe 2p was realized for PANI:PSS/SWCNT/Fc and is presented in Figure 6j. The results show that the Fe is present in Fe^2+^ and Fe^3+^ states in this layer, since the most stable oxidation states of the iron centre in ferrocene are +2 and +3. Table 4 shows the Fe 2p component for the new layer.

In addition, XRD was used to study the crystalline phase, as shown in Figure S6. Firstly, it can be observed that PANI exhibits multiple diffraction peaks. Then, the initial ferrocene presents narrow peaks which confirms the good crystal quality. However, in the compound PANI:PSS/SWCNT/Fc, the corresponding peaks for ferrocene are absent. Taking into account the small signal for Fe 2p in XPS, most probably, the ferrocene concentration is small, and cannot be identified in X-ray diffraction experiments. The broad feature located at 21.31° indicates the presence of the small SWCNTs domains with the interplanar distance, d of 0.41 nm, according to the Bragg’s law: 2dsinθ=λ, where **2**θ is the angular position and λ is the wavelength of the incident X-rays. Bashkin et al. reported an interplanar distance of SWCNTs of 0.46 nm [48], whereas other researchers reported smaller values [49,50,51], closely to graphite (0.34 nm). Further, an evaluation of the size of the crystalline domains was provided with the Scherrer equation, that relates the full width half maximum (FWHM) of the Bragg peak by the mean crystallite size [48]. It was found that the SWCNTs have small crystalline domains with a size of 1.5 nm. Other peaks can be identified as cubic gold with an ICDD card no. 004-0784 as (111), (200), (220), and (311) reflections.

### 3.2. Electrochemical Characterizations

#### 3.2.1. PANI Films Formation

**Figure 7:**
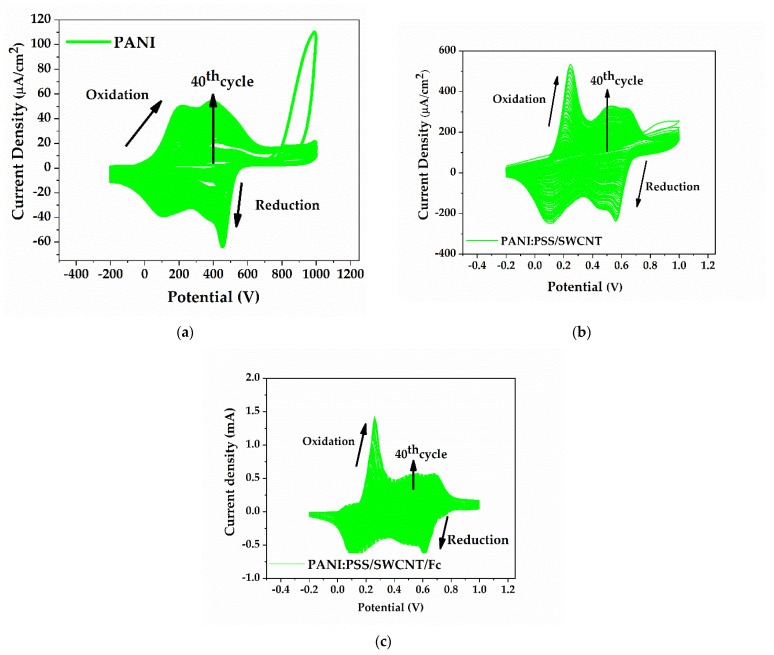
Cyclic voltammograms during formation of (**a**) PANI, (**b**) PANI:PSS/SWCNT, (**c**) PANI:PSS/SWCNT/Fc; For each CV measurement, n = 40 cycles were taken from −0.2 V to 0.9 V vs. Ag/AgCl with a scan rate of 50 mV/s.

The CVs during 40 cycles synthesis of PANI were recorded and are shown in Figure 7a. The first oxidation peaks at about 0.20 V were due to the transformation of PANI from a fully reduced state to a neutral state. The oxidation peaks at about 0.40 V show the transformation from emeraldine salt to the pernigraniline form. After 40 cycles of EP, the oxidation peaks currents increased, indicating that a PANI film with high conductivity had been deposed onto the IDE area.

In accordance with other research, polyaniline in a sulphonated form showed a structure as the ion conductive polymers [52]. In emeraldine salt, the H_2_SO_4_ species are ionically bonded with -NH groups from the polymer structure, but these bonds are very weak and it is necessary to supplement doping the polyaniline to improve its electrical conduction. Figure S7 shows the chemical structure of PANI in a sulphuric acidic medium. Adding the optimum SWCNT ensures the charge transfer efficiency and therefore better conductivity of the film. 

#### 3.2.2. PANI:PSS/SWCNT Films Formation

Figure 7b shows the formation of PANI:PSS/SWCNT, demonstrated by the small oxidation peaks at about 0.50 V and by a shift of the PANI oxidation peak from 0.4 to 0.6 V. The small peaks appeared due to the branched structure of PANI:PSS/SWCNT (this has also been demonstrated by Dai Tran et al.) [53]. Maintaining the same experimental conditions, the current intensity of PANI:PSS/SWCNT was almost 15 times larger than the simple PANI, which proves the increase of the conductivity and also the surface-active area. Therefore, adding the SWCNT in the EP solution led to an increase in the electroactivity of the IDEs. These are the most important parameters for sensor performance.

#### 3.2.3. PANI:PSS/SWCNT/Fc Films Formation

The formation of PANI:PSS/SWCNT/Fc was made by 40 cycles of EP on the IDEs and it obtained an enhanced current response, as shown in Figure 7c. This was possible through a π-π stacking interaction between Fc and SWCNT and was due to the nanohybrid film formation. Therefore, the addition of Fc led to a better electroactivity of the layer. This observation was also made by A. Kumar et al. in regard to the SWCNT/Fc noncovalent interaction [54]. A small additional peak was also observed at about 0.080 mV, which was made possible due to some ionic interaction of Fc with PANI. When the IDEs with PANI:PSS/SWCNT/Fc were immersed in PBS (pH 7.0) with 0.1 M KCl for redox characterization, the Fc bonding was stronger.

**Figure 8:**
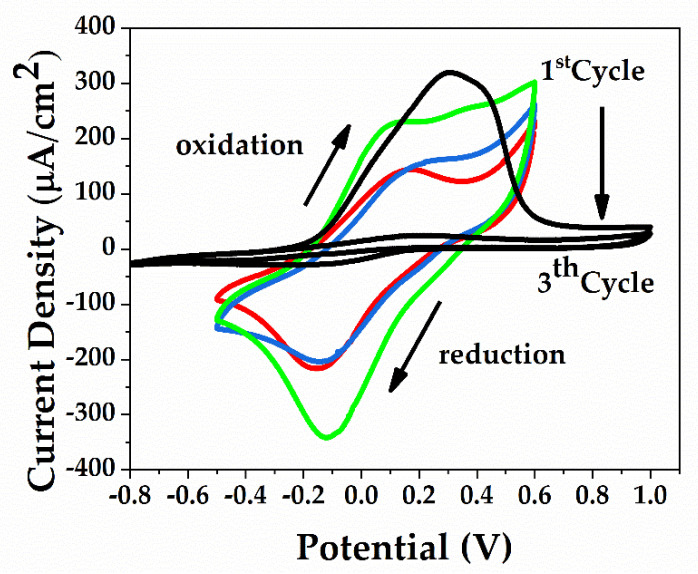
CV curves recorded in phosphate buffer saline PBS (7.00) and 0.1 M KCl of the EP/IDEs with the PANI:PSS/SWCNT/Fc film compared to IDEs with PANI:PSS/SWCNT.

Figure 8 shows the electroactive behavior of the film due to the redox couple Fc/Fc+ (ferrocene/ferrocenium), presented by the new EP/IDEs with the PANI:PSS/SWCNT/Fc film compared to the IDEs with PANI:PSS/SWCNT (without Fc). Therefore, the Fc addition showed an enhanced electron transfer process to the IDEs. Initially, the PANI:PSS/SWCNT without Fc has a conductive behavior with a higher peak at about 0.28 V that corresponds to the first oxidation peak of PANI in the PBS (KCl 0.1 M) electrolyte at pH 7. Following the cycles, this layer becomes insulating, while the ferrocene layer shows in the first cycles the oxidation-reduction due the presence of Fc in the layer and then its redox activity begins to decrease (the green curve to red curve decrease the conductivity).

#### 3.2.4. EIS Results

**Figure 9:**
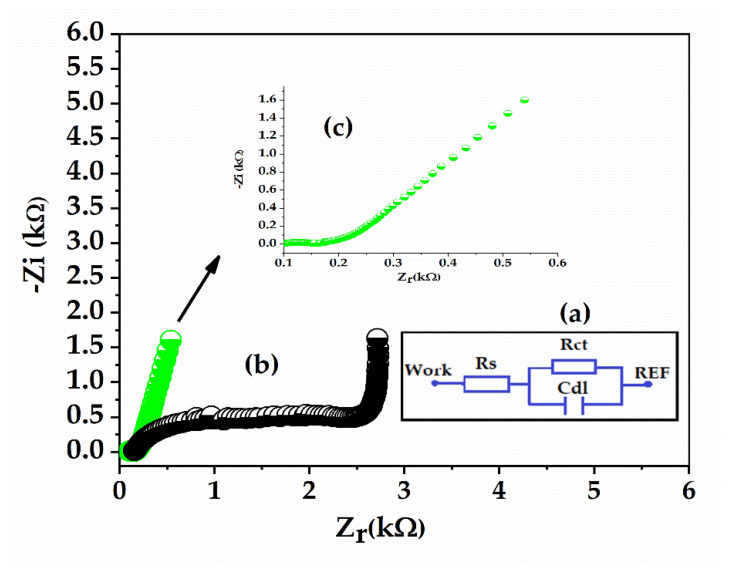
(**a**) Equivalent circuit for fitting EIS results with the solution resistance (R_s_), double layer capacitance (C_dl_) and the electron transfer resistance (R_ct_) (**b**) Nyquist plot (-Z_i_ vs Z_r_) for PANI:PSS/SWCNT/Fc (green) and PANI:PSS/SWCNT (black) (**c**) Nyquist plot (-Z_i_ vs Z_r_) for PANI:PSS/SWCNT/Fc (green) at a lower scale.

EIS is a useful tool for examining the surface modification of the sensor after the deposition of PANI:PSS/SWCNT composites without Fc comparative with Fc. If the sensors are EIS characterized in the presence of [Fe (CN) 6]^3^^−^^/4−^ redox probe the results can be can be interpreted by recording the Nyquist Plot (−Zi vs. Zr) for both layers and fitted with an equivalent circuit presented in the inset figure from Figure 9a, which includes the solution resistance (R_s_), double layer capacitance (C_dl_), and the electron transfer resistance (R_ct_). The “Auto. R_1_R_2_C Fitting” method is used to fit the curve to a circle while running the sequence.

As shown in Figure 9b, the Nyquist plots of PANI:PSS/SWCNT were almost semi-circular in the high frequency range and formed a straight line over the entire frequency range for the PANI:PSS/SWCNT/Fc film (Figure 9c). The verticality of the film with Fc was clearly better, therefore demonstrating a lower resistance Rct for the Fc film compared to Rct when Fc was absent. These results came upon completion of the CV results, where a current increase response when the Fc was incorporated in the PANI:PSS/SWCNT was observed.

### 3.3. Wearable and Additional Applications

#### 3.3.1. CO dc Measurements

- Humidity studies

**Figure 10:**
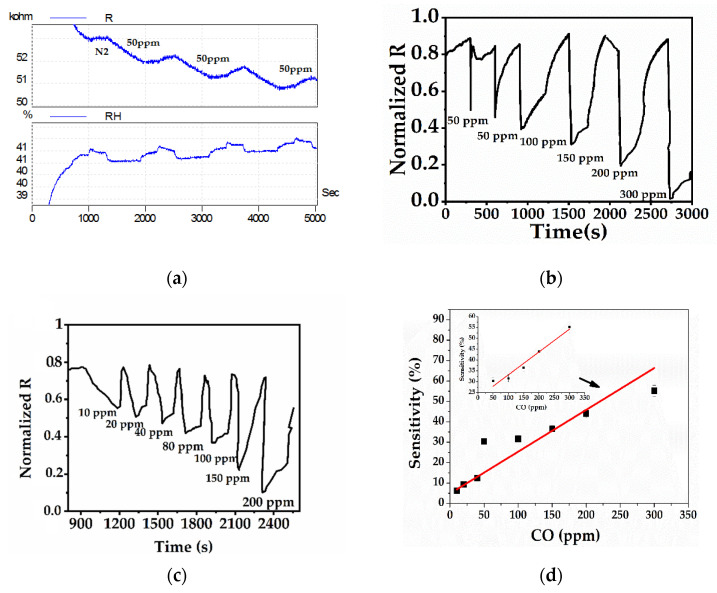
(**a**) The sensor behavior at a 40% relative humidity after three 50 ppm consecutive concentrations; (**b**) normalized values of resistances of sensor response [0,1] at low CO concentrations; (**c**) normalized values of resistances of sensor response [0,1] at high CO concentrations; (**d**) sensitivity variation of PANI:PSS/SWCNT/Fc with CO.

The IDEs prepared with PANI:PSS/SWCNT and PANI:PSS/SWCNT/Fc were tested for humidity. The air resistances were registered at a temperature of 23 °C with approximately 40% relative humidity. It is known from the literature that when two materials are used separately to determine the humidity, the resistance of the SWCNT layer exposed to water increases and the one with PANI decreases [27,55]. It is this opposite electrical response that can lead to avoiding humidity if the number of SWCNT-related PANI molecules is optimized by varying the number of cycles. Thus, after 20 and 40 cycles, the values of air and nitrogen resistances, respectively were recorded. The aim was to evaluate the difference between the sensor-measured resistance in air and in nitrogen (ΔR) at a constant concentration of Fc in the EP solution at a different CV cycle. The results showed that the resistance in air was different for each layer and depended on the CV cycles. If the Fc layer was formed at 40 cycles in the S4 solution on the IDEs, resistance was eliminated or became negligible. An explanation for these results may involve an increase of the electroactivity of PANI when noncovalent Fc is added to the layer (as shown in the electrochemical results). The Fc may determine the doping of PANI or may donate electrons to charge carriers holes of p-type SWCNT, so the normal donation of electrons by water molecules to SWCNT are hindered [27]. The initial values (without CO) for ΔR (difference between resistance in nitrogen and resistance in air) are shown in Table 5:

When consecutively adding 50 ppm CO at a 41% relative humidity, the sensor showed a decrease of resistance each time. This confirms its reproducibility in humid conditions, as shown in Figure 10a. The relative humidity changes were minimal at about 1%. Two ranges of concentrations were analyzed: Low concentration between 0–10–20–40–80 ppm and high concentrations between 50–100–150–200–300 ppm. Both values of resistances (normalized [0,1]) showed a decrease of concentrations in the presence of CO (Figure 10b,c). Figure 10d shows the sensitivity variation of PANI:PSS/SWCNT/Fc with CO.

#### 3.3.2. Analytical Parameters of the Sensor

The most important parameters of a gas sensor are sensitivity, recovery time, gas selectivity, and detection limit.

Two ranges of concentrations were analyzed: Low concentration between 0–10–20–40–80 ppm and high concentrations between 50–100–150–200–300 ppm. In both cases, the resistances decrease with the increase of the CO concentrations (Figure 10b,c).

As presented in Figure 10b, the sensor had a rapid response when exposed to CO but then showed a little increase in resistance after eliminating CO and stabilization. Under nitrogen exposure, the sensor’s resistance increased at its initial value. Considering the morphological, structural, and electrical characterizations of the PANI:PSS/SWCNT/Fc layer, a possible reaction mechanism between the layer and CO may be represented by the fact that after the COs physical absorption onto the sensor surface, a charge transfer occurred between the SWCNT and PANI that may be enhanced by the addition of Fc as a mediator. Therefore, the conductivity of the composite material was significantly increased. After removing the CO, the sensor returned to its initial state.

Figure 10d shows the sensitivities of the PANI:PSS/SWCNT/Fc layer using Equation (1) in the presence of five different CO concentrations. The sensor’s sensitivity increased with the increase of CO concentration. The sensor showed a good sensitivity for all concentrations at room temperature with approximatively 6% for the first level CO concentration of 10 ppm. According to the calibration curve from Figure 10d, the estimated limit of detection (LOD) for CO (LOD = $\frac{3S_{D}}{m}$ where m is the slope of the linear part and $S_{D}$ is the standard deviation of noise in the response curve) was determined to be 40 ppm for concentrations ranging between 40 and 300 ppm. After fitting, the linearity for concentrations between 5 and 300 ppm was lower (R^2^ = 0.890) than for those between 40 and 300 ppm (R^2^ = 0.970). The calibration curve equations for both concentration ranges and limits of detection are shown in Table 6.

**Figure 11:**
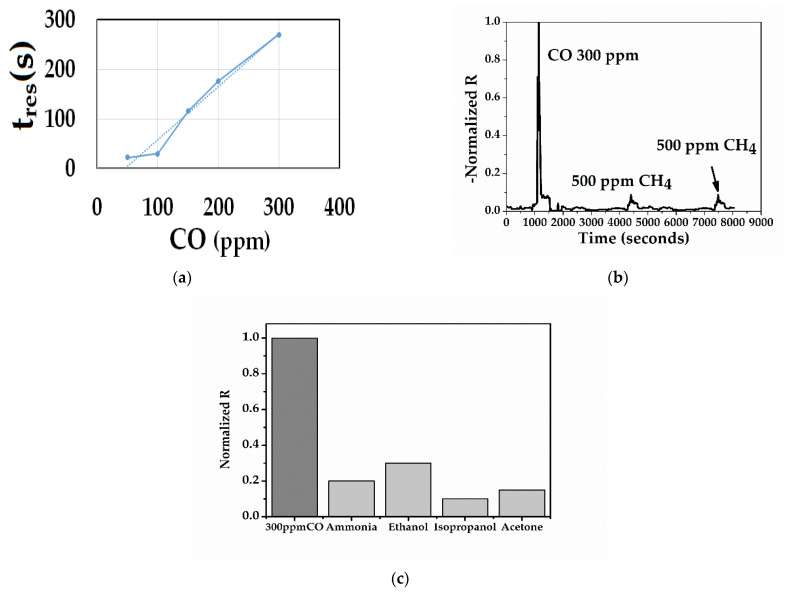
(**a**) Response time of the sensor at 50, 100, 150, 200, and 300 ppm CO; (**b**) selectivity of PANI:PSS/SWCNT/Fc in the presence of 500 ppm methane (at room temperature in the presence of dry air (N_2_ and O_2_); (**c**) selectivity of PANI:PSS/SWCNT/Fc (at room temperature in the presence of 100 ppm NH3, 2000 ppm ethanol, 1000 ppm acetone, and 200 ppm isopropanol).

Response time (**t_res_**) is the time required to reach 90% of the signal of a constant response and recovery time (**t_rec_**) is the time required to restore 90% of the baseline of the initial resistance before the sensor is exposed to gas. Figure 11a clearly shows that the response time of the sensor after exposure at a concentration of 50 ppm CO was rapid, from 297 to 327 s, the decrease being sudden. The average response time for three measurements after sensor exposure to 100 ppm CO was 30 s. The average response time of the sensor after desorption of 100 ppm CO was 130 s. All the response times of the sensor increased with all CO concentrations and were much lower than the recovery times. For the sensing mechanism of PANI:PSS/SWCNT/Fc, the fitted model of the 1st Langmuir Adsorption-desorption was used and presented in Supplementary (Figure S8).

Selectivity is defined as the ratio of the target gas response to another gas or other compound that may interfere with it. Generally, a PANI-based sensor may interfere with alcohols, ammonia, and other gases including methane, CO_2_, and NO_2_ [56]. Figure 11b clearly shows that the PANI:PSS/SWCNT/Fc exhibited a higher response for 300 ppm CO among methane. The responses of normalized resistance after exposure to alcohols were lower than the normalized resistance after exposure to 300 ppm of CO (Figure 11c).

The variance in the concentrations obtained by our layer was evaluated by the relative standard deviation percentage (RSD) between replicates that were calculated by Equation (3), where S is the standard error deviation of the blanks and $\bar{X}$ is the average of the resistance measurements. Under optimal conditions, the layer was deposed by CV on IDEs and were tested independently for the following CO concentrations: 50, 100, and 300 ppm. The RSD values for each sensor at 50 ppm exhibited a much higher error (5%) than the RSDs obtained at 100 and 300 ppm, with an average of three RSD values of about 3.14%. However, these values were acceptable from an analytical point of view. The higher errors were generally reported at lower concentrations.

(3) $$\mathrm{RSD}\;=\;\frac{s}{\bar{X}}*100$$

Considering the thermal stability of PANI up to temperatures of 100°, in the future this time could be shortened by a possible heating of it. Moreover, other researchers have found accelerating desorption and shortening the recovery time with temperature after heating the PANI-based sensor [57].

#### 3.3.3. Wearable Potential Development

**Figure 12:**
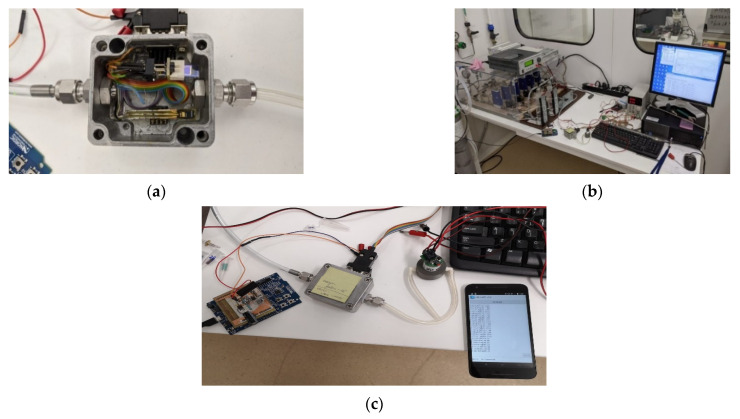
(**a**) Test chamber in which the sensor is placed to expose the known CO gas concentrations; (**b**) laboratory setup used to test the sensor’s correct operation and also to verify our portable readout electronics; (**c**) readout module connected to the sensor in the test chamber, with a smartphone wireless connection established for data logging.

Using the electronic readout test setup presented in Figure 12, the developed sensors were measured in the laboratory, in a controlled test chamber. In addition, the results were compared with the portable electronics and the laboratory equipment setup.

Figure 12a shows the sensor placed in the test chamber where a gas mixture with a known CO concentration was introduced. The sensor was first monitored using laboratory apparatus in order to measure resistance. Subsequently, the sensor readout was performed using the developed test platform. The correlation between the laboratory equipment data and the portable platform developed for the application was very good: The differences between the two measurements were lower that 5%.

The experimental setup with SMU and PC data is shown in Figure 12b, while the sensor in the test chamber connected to the test platform is presented in Figure 12c.

## 4. Conclusions

The electroactive performance of a new PANI:PSS/SWCNT/Fc hybrid layer obtained by EP was described and evaluated for CO detection. The hybrid layer shows mechanical and environmental stability and can be customized for a very good response to CO detection. Although humidity poses a problem since PANI is hydrophilic, our results show that humidity would be almost negligible if the proper parameters were chosen for the layer electrosynthesis: Electropolymerization cycles and Fc concentrations. The layer offers a rapid response and recovery cycle in the presence of various CO concentrations. Since the sensitivity was better than other similar layers based on PANI/CNT, further experiments will target a broader range of CO concentrations and a shortening of the recovery times.

During the experiments, the layer was optimized to obtain proper and reproducible resistance, demonstrating that it can further be successfully applied as a material for the miniaturized wearable gas sensor. The PANI:PSS/SWCNT/Fc-based sensor may be used as a proper material for CO detection and could be optimized further for various wearable applications.

## Figures and Tables

**Table 1:** The component for C1s spectra.

Component	C-C, C=C, C-H	C-O, C-N	C=O	O-C=O	Aromatic
PANI: PSS/SWCNT:Fc	79.07	17	1.44	0.9	1.6
PANI:PSS/SWCNT	83.77	12.98	0.47	0.34	2.45
PANI	56.68	17.78	7.71	11.35	6.48

**Table 2:** The component for O1s.

Component	C=O	C-O	C-O-C
PANI:PSS/SWCNT:Fc	13.54	34	52.45
PANI:SWCNT	8.57	51.8	39.64
PANI	15.24	29.62	55.14

**Table 3:** The component for N1s.

Component	C-N+	C-N=	C-NH-
PANI:PSS/SWCNT/Fc	10.39	47.14	42.46
PANI:SWCNT	6.7	20.92	72.38
PANI	0	77.65	22.35

**Table 4:** The component for Fe 2p.

Component	C-N+	C-N=	C-NH-
PANI:PSS/SWCNT/Fc	10.39	47.14	42.46
PANI:SWCNT	6.7	20.92	72.38
PANI	0	77.65	22.35

**Table 5:** ΔR values for PANI:PSS/SWCNT and PANI:PSS/SWCNT/Fc at 20 and 40 CV cycles.

Layer	Cycles	Fc (mM)	ΔR (kΩ)
PANI:PSS/SWCNT	20	-	160
	40	-	107
PANI:PSS/SWCNT/Fc	20	1.33	12
	40	1.33	2

**Table 6:** Analytical parameters from calibration curves.

Sensor Layer	Range	Equation	LOD	R^2^
PANI:PSS/SWCNT/Fc	5–300 ppm	S(%) = (10.610 ± 3.520) + (0.160 ± 0.020) $*\;C_{CO}$(ppm)	66 ppm	0.890
PANI:PSS/SWCNT/Fc	40–300 ppm	S(%) = (23.810 ± 1.560) + (0.100 ± 0.009) $*\;C_{CO}$(ppm)	40 ppm	0.970

## Data Availability

Not applicable.

## Supplementary Materials

The following are available online at https://www.mdpi.com/1424-8220/21/5/1801/s1, Figure S1: (a) Layout interdigitated sensor, (b) Area with interdigitated electrodes, Figure S2: Diagram with measuring instalation. (a) CO cylinder, (b) N_2_ cylinder, (c) Mass flow controller for CO, (d) Mas flow controller for N_2_, (e) Gas mixing compartment, (f) GID sensor used for CO testing, (g) Source and multimeter, (h) Software, Figure S3: (a) PANI/SWCNT, (b) PANI:PSS(3.2%)/SWCNT, Figure S4: Sensors with 0, 6, 8, 15, 25, 40 and 70 CV cycles, Figure S5: XPS survey spectra of the: (a) PANI; (b) PANI:PSS/SWCNT; (c) PANI:PSS/SWCNT/Fc, Figure S6: XRD for PANI, Ferrocene and PANI:PSS:SWCNT:Fc, Figure S7: Polyaniline as Emeraldine salt, in sulfuric acid media, Figure S8: (a) Fitted response and recovery curve of PANI:PSS/SWCNT/Fc in presence of 300 ppm CO; (b) logharitmic dependence of ln (1-Normalized ΔG) and (c) logharitmic dependence of Normalized ΔG to obtain 1/t_res_ and respectivelly 1/t_rec_.

## References

[1] Kim S.G., Jun J., Lee J.S., Jang J. (2019). A highly sensitive wireless nitrogen dioxide gas sensor based on an organic conductive nanocomposite paste. J. Mater. Chem. A.

[2] Zampetti E., Pantalei S., Muzyczuk A., Bearzotti A., De Cesare F., Spinella C., Macagnano A. (2013). A high sensitive NO_2_ gas sensor based on PEDOT–PSS/TiO_2_ nanofibres. Sens. Actuators B Chem..

[3] Park S.J., Lee S.H., Yang H., Park C.S., Lee C.S., Kwon O.S., Jang J. (2016). Human dopamine receptor-conjugated mul-tidimensional conductive polymer nanofiber membrane for dopamine detection. ACS Appl. Mater. Inter..

[4] Oh J., Kim Y.K., Lee J.S., Jang J. (2019). Highly porous structured polyaniline nanocomposites for scalable and flexible high-performance supercapacitors. Nanoscale.

[5] Wu Z.-S., Parvez K., Li S., Yang S., Liu Z., Liu S., Feng X., Müllen K. (2015). Alternating Stacked graphene-conducting polymer compact films with ultrahigh areal and volumetric capacitances for high-energy micro-supercapacitors. Adv. Mater..

[6] Lee K., Cho K.H., Ryu J., Yun J., Yu H., Lee J., Jang J. (2017). Low-cost and efficient perovskite solar cells using a surfac-tant-modified polyaniline: Poly (styrenesulfonate) hole transport material. Electrochi. Acta..

[7] Huang H., Yao J., Chen H., Zeng X., Chen C., She X., Li L. (2016). Facile preparation of halloysite/polyaniline nanocomposites via in situ polymerization and layer-by-layer assembly with good supercapacitor performance. J. Mater. Sci..

[8] Liu Q., Wu J., Lan Z., Zheng M., Yue G., Lin J., Huang M. (2014). Preparation of PAA-g-PEG/PANI polymer gel electrolyte and its application in quasi solid state dye-sensitized solar cells. Polym. Eng. Sci..

[9] Gao J., Yang Y., Zhang Z., Yan J., Lin Z., Guo X. (2016). Bifacial quasi-solid-state dye-sensitized solar cells with Poly (vinyl pyrrolidone)/polyaniline transparent counter electrode. Nano Energy.

[10] Lee K., Yu H., Lee J.W., Oh J., Bae S., Kim S.K., Jang J. (2018). Efficient and moisture-resistant hole transport layer for inverted perovskite solar cells using solution-processed polyaniline. J. Mater. Chem. C.

[11] Lee H.J., Anoop G., Lee H.J., Kim C., Park J.W., Choi J., Kim Y.M. (2016). Enhanced thermoelectric performance of PEDOT: PSS/PANI–CSA polymer multilayer structures. Energy Environ. Sci..

[12] Han H., Lee J.S., Cho S. (2019). Comparative studies on two-electrode symmetric supercapacitors based on polypyrrole:Poly(4-styrenesulfonate) with different molecular weights of poly(4-styrenesulfonate). Polymers.

[13] Cho S., Kim M., Jang J. (2015). Screen-printable and flexible RuO2Nanoparticle-decorated PEDOT:PSS/Graphene nanocomposite with enhanced electrical and electrochemical performances for high-capacity supercapacitor. ACS Appl. Mater. Interfaces.

[14] Cho S., Lee J.S., Jun J., Kim S.G., Jang J. (2014). Fabrication of water-dispersible and highly conductive PSS-doped PANI/graphene nanocomposites using a high-molecular weight PSS dopant and their application in H2S detection. Nanoscale.

[15] Rosa T., Aroeira G.J., Parreira L.S., Codognoto L., Santos M.C., Simões F.R. (2015). Self-assembled films based on polyani-line/multiwalled carbon nanotubes composites and sulphonated polystyrene deposited onto ITO substrates. Synthetic Met..

[16] Sarvi A., Sundararaj U. (2014). Electrical permittivity and electrical conductivity of multiwall carbon nanotube-polyaniline (MWCNT-PANi) core-shell nanofibers and MWCNT-PANi/polystyrene composites. Macromol. Mater. Eng..

[17] El Khakani M.A., Yi J.H. (2004). The nanostructure and electrical properties of SWNT bundle networks grown by an all-laser growth process for nanoelectronic device applications. Nanotechnology.

[18] Bernholc J., Brenner D.W., Nardelli M.B., Meunier V., Roland C. (2002). Mechanical and electrical properties of nanotubes. Annu. Rev. Mater. Res..

[19] Fan Z., Wei T., Luo G., Wei F. (2005). Fabrication and characterization of multi-walled carbon nanotubes-based ink. J. Mater. Sci..

[20] Spitalsky Z., Tasis D., Papagelis K., Galiotis C. (2010). Carbon nanotube–polymer composites: Chemistry, processing, mechanical and electrical properties. Prog. Polym. Sci..

[21] Kharisov B.I., Kharissova O.V., Méndez U.O. (2014). Methods for dispersion of carbon nanotubes in water and common sol-vents. MRS Online Proc. Libr. Arch..

[22] Kulkarni M.V., Apte S.K., Naik S.D., Ambekar J.D., Kale B.B. (2013). Ink-jet printed conducting polyaniline based flexible humidity sensor. Sens. Actuators B Chem..

[23] Zeng F.-W., Liu X.-X., Diamond D., Lau K.T. (2010). Humidity sensors based on polyaniline nanofibres. Sens. Actuators B Chem..

[24] Farahani H., Wagiran R., Hamidon M.N. (2014). Humidity sensors principle, mechanism, and fabrication technologies: A com-prehensive review. Sensors.

[25] Fratoddi I., Venditti I., Cametti C., Russo M.V. (2015). Chemiresistive polyaniline-based gas sensors: A mini review. Sens. Actuators B Chem..

[26] Sengupta P.P., Barik S., Adhikari B. (2006). Polyaniline as a Gas-Sensor Material. Mater. Manuf. Process..

[27] Zhang T., Mubeen S., Yoo B., Myung N.V., Deshusses M.A. (2009). A gas nanosensor unaffected by humidity. Nanotechnology.

[28] Shimizu Y., Yamamoto S., Takase S. (2017). A thick-film impedancemetric carbon monoxide sensor using layered perovskite-type cuprate. Sens. Actuators B Chem..

[29] Radhakrishnan S., Paul S. (2007). Conductive polypyrrole modified with ferrocene for applications in carbon monoxide sen-sors. Sens. Actuators B Chem..

[30] Yang X., Lu Y., Ma Y., Li Y., Du F., Chen Y. (2006). Noncovalent nanohybrid of ferrocene with single-walled carbon nanotubes and its enhanced electrochemical property. Chem. Phys. Lett..

[31] Guldi D.M., Marcaccio M., Paolucci D., Paolucci F., Tagmatarchis N., Tasis D., Prato M. (2003). Single-wall carbon nano-tube–ferrocene nanohybrids: Observing intramolecular electron transfer in functionalized SWNTs. Angew. Chem. Int. Edit..

[32] Zhang Y., D’Ambra C.A., Katsumata R., Burns R.L., Somervell M.H., Segalman R.A., Bates C.M. (2019). Rapid and se-lective deposition of patterned thin films on heterogeneous substrates via spin coating. ACS Appl. Mater. Inter..

[33] Hu Z., Zhang J., Xiong S., Zhao Y. (2012). Performance of polymer solar cells fabricated by dip coating process. Sol. Energy Mater. Sol. Cells.

[34] Kovacik P., Willis S.M., Matichak J.D., Assender H.E., Watt A.A. (2012). Effect of side groups on the vacuum thermal evap-oration of polythiophenes for organic electronics. Org. Electron..

[35] Park J.Y., Advincula R.C. (2011). Nanostructuring polymers, colloids, and nanomaterials at the air–Water interface through Langmuir and Langmuir–Blodgett techniques. Soft Matter..

[36] Tekin-Kazancioglu E.E., Smith P.J., Schubert U.S. (2008). Inkjet printing as a deposition and patterning tool for polymers and inorganic particles. Soft Matter..

[37] Mihailescu C.M., Moldovan C.A., Brasoveanu C., Savin M., Dinulescu S., Firtat B., Stanciu I. (2019). Polyaniline Working Electrodes For Glucose Sensing. CAS.

[38] Ansari R. (2006). Polypyrrole conducting electroactive polymers: Synthesis and stability studies. J. Chem..

[39] Fan L.-Z., Maier J. (2006). High-performance polypyrrole electrode materials for redox supercapacitors. Electrochem. Commun..

[40] Kim S., Jang L., Park H.S., Lee J.Y. (2016). Electrochemical deposition of conductive and adhesive polypyrrole-dopamine films. Sci. Rep..

[41] Liu C., Hayashi K., Toko K. (2010). Electrochemical deposition of nanostructured polyaniline on an insulating substrate. Electrochem. Commun..

[42] Rodgers M.M., Alon G., Pai V.M., Conroy R.S. (2019). Wearable technologies for active living and rehabilitation: Current research challenges and future opportunities. J. Rehabil. Assist. Technol. Eng..

[43] Park J.H., Seo J., Kim C., Joe D.J., Lee H.E., Im T.H., Seok J.Y., Jeong C.K., Ma B.S., Park H.K. (2018). Flash-induced stretchable cu conductor via multiscale-interfacial couplings. Adv. Sci..

[44] Serrano-Garcia W., Jayathilaka W.A.D.M., Chinnappan A., Tran T.Q., Baskar C., Thomas S.W., Ramakrishna S. (2019). Nanocomposites for electronic applications that can be embedded for textiles and wearables. Sci. China Ser. E Technol. Sci..

[45] Kuzmany H., Shi L., Kürti J., Koltai J., Chuvilin A., Saito T., Pichler T. (2017). The growth of new extended carbon nanophases from ferrocene inside single-walled carbon nanotubes. Phys. Status Solidi Rapid Res. Lett..

[46] Kumar R., Mitra A., Varma G.D. (2019). Study of vortex glass-liquid transition in superconducting Fe(Te, Se) thin films on LaAlO_3_ substrates. J. Appl. Phys..

[47] Tantawy H.R., Kengne B.-A.F., McIlroy D.N., Nguyen T., Heo D., Qiang Y., Aston D.E. (2015). X-ray photoelectron spectroscopy analysis for the chemical impact of solvent addition rate on electromagnetic shielding effectiveness of HCl-doped polyaniline nanopowders. J. Appl. Phys..

[48] Bashkin I.O., Antonov V.E., Bazhenov A.V., Bdikin I.K., Borisenko D.N., Krinichnaya E.P., Moravsky A.P., Harkunov A.I., Shul’Ga Y.M., Ossipyan Y.A. (2004). Thermally stable hydrogen compounds obtained under high pressure on the basis of carbon nanotubes and nanofibers. J. Exp. Theor. Phys. Lett..

[49] Kawasaki S., Matsuoka Y., Yokomae T., Nojima Y., Okino F., Touhara H., Kataura H. (2005). XRD and TEM study of high pressure treated single-walled carbon nanotubes and C60-peapods. Carbon.

[50] Futaba D.N., Yamada T., Kobashi K., Yumura M., Hata K. (2011). Macroscopic wall number analysis of single-walled, double-walled, and few-walled carbon nanotubes by X-ray diffraction. J. Am. Chem. Soc..

[51] Pierard N., Fonseca A., Colomer J.-F., Bossuot C., Benoit J.-M., Van Tendeloo G., Pirard J.-P., Nagy J. (2004). Ball milling effect on the structure of single-wall carbon nanotubes. Carbon.

[52] Hasan S.M., Hussein Z.A.A. (2014). The effect of H_2_SO_4_ acid as a doping agent on the structure of Polyaniline prepared at room temperature. Int. J. Appl. Innov. Eng. Manag..

[53] Tran L.D., Nguyen D.T., Nguyen B.H., Do Q.P., le Nguyen H. (2011). Development of interdigitated arrays coated with functional polyaniline/MWCNT for electrochemical biodetection: Application for human papilloma virus. Talanta.

[54] Kumar A., Kumar V., Awasthi K. (2018). Polyaniline–Carbon nanotube composites: Preparation methods, properties, and appli-cations. Polym. Plast Technol..

[55] Jain S., Chakane S., Samui A.B., Krishnamurthy V.N., Bhoraskar S.V. (2003). Humidity sensing with weak acid-doped poly-aniline and its composites. Sens. Actuat B Chem..

[56] Roy A., Ray A., Sadhukhan P., Naskar K., Lal G., Bhar R., Sinha C., Das S. (2018). Polyaniline-multiwalled carbon nanotube (PANI-MWCNT): Room temperature resistive carbon monoxide (CO) sensor. Synth. Met..

[57] Kukla A., Shirshov Y., Piletsky S. (1996). Ammonia sensors based on sensitive polyaniline films. Sens. Actuators B Chem..

