# Supplementary material for: A New Hybrid Sensitive PANI/SWCNT/Ferrocene-Based Layer for a Wearable CO Sensor
Source: Sensors (Basel). 2021 Mar 5;21(5):1801. doi: 10.3390/s21051801 (PMC7961761; doi:10.3390/s21051801)
Supplement: Supplementary file 1 [file sensors-21-01801-s001.pdf]

# Supplementary Material

## A new hybrid sensitive PANI/SWCNT/Ferrocene-based layer for a wearable CO sensor

**Mihaela Savin [1, 2], Carmen-Marinela Mihailescu [1*], Viorel Avramescu[1], Silviu Dinulescu[1], Bogdan Firtat[1], Gabriel Craciun[1], Costin Brasoveanu[1], Cristina Pachiu[1], Alina Catrinel Ion[2], Cosmin Romanitan[1], Andreea-Bianca Serban[3,4], Carmen Moldovan[1*]**

[1]National Institute for Research and Development in Microtechnologies, Erou Iancu Nicolae Street 126A, Bucharest, 077190, Romania; mihaela.savin@imt.ro (M.S.); viorel.avramescu@imt.ro (V.A.); silviu.dinulescu@imt.ro (S.D.); bogdan.firtat@imt.ro (B.F.); gabriel.craciu@imt.ro (G.C.); costin.brasoveanu@imt.ro (C.B.); cristina.pachiu@imt.ro (C.P.); cosmin.romanitan@imt.ro (C.R)

[2] University Politehnica of Bucharest, Department of Applied Chemistry and Materials Science, 1-7 Polizu, 011061 Bucharest, Romania. alina.ion@upb.ro (A.C.I.)

[3] Doctoral School in Engineering and Applications of Lasers and Accelerators, University Politehnica of Bucharest, 060042 Bucharest, Romania. andreea.serban@eli-np.ro (A.B.S)

[4] Extreme Light Infrastructure-Nuclear Physics (ELI-NP), 'Horia Hulubei' National R&D Institute for Physics and Nuclear Engineering (IFIN-HH), 30 Reactorului Street, 077125 Măgurele, Ilfov, Romania (A.B.S)

* Correspondence: carmen.mihailescu@imt.ro (C.M.M) and carmen.moldovan@imt.ro (C.M.)

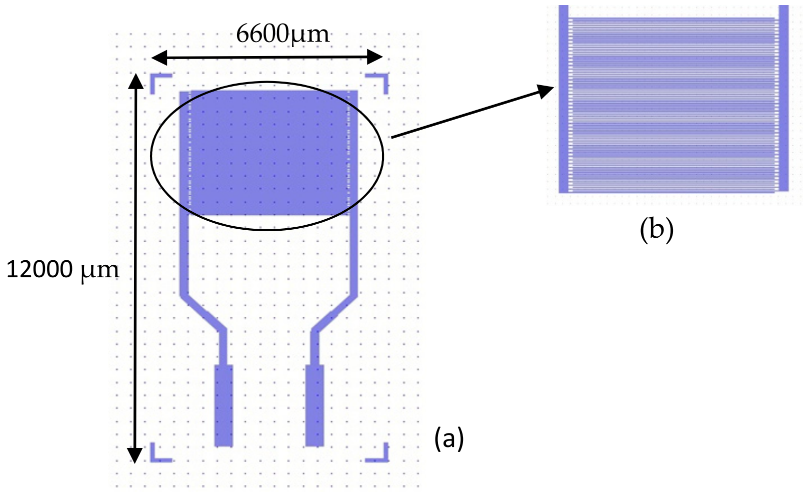


**Figure S1.** (a) Layout interdigitated sensor, (b) Area with interdigitated electrodes.

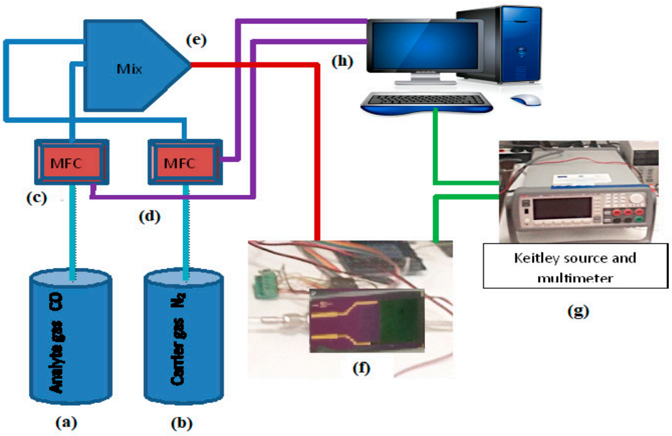


**Figure S2.** Diagram with measuring instalation. (a) CO cylinder, (b) $N_2$ cylinder, (c) Mass flow controller for CO, (d) Mas flow controller for $N_2$, (e) Gas mixing compartment, (f) GID sensor used for CO testing, (g) Source and multimeter, (h) Software.

(a) 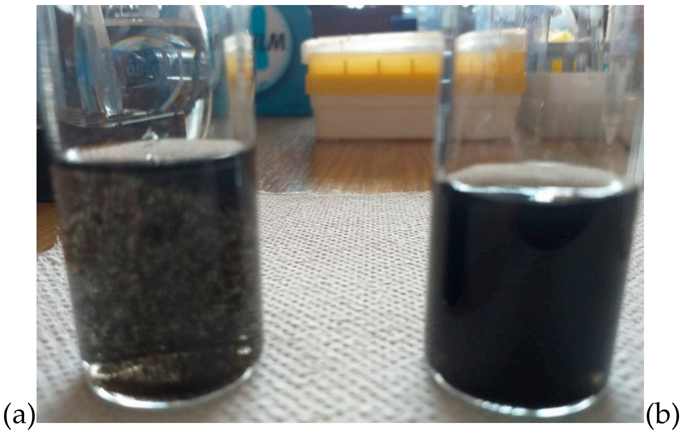 (b)

**Figure S3**. (a) PANI/SWCNT, (b) PANI:PSS(3.2%)/SWCNT

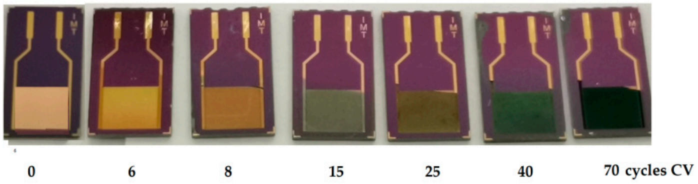


**Figure S4. Sensors with 0, 6, 8, 15, 25, 40 and 70 CV cycles.**

**Figure S5.** XPS survey spectra of the: (a) PANI ; (b) PANI:PSS/SWCNT; (c)PANI:PSS/SWCNT/Fc;

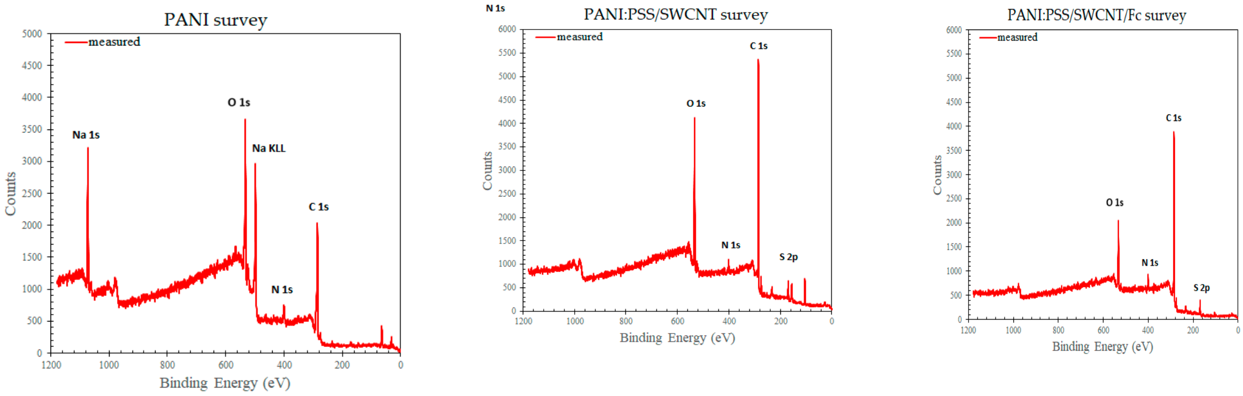


**(a)** **(b)** **(c)**

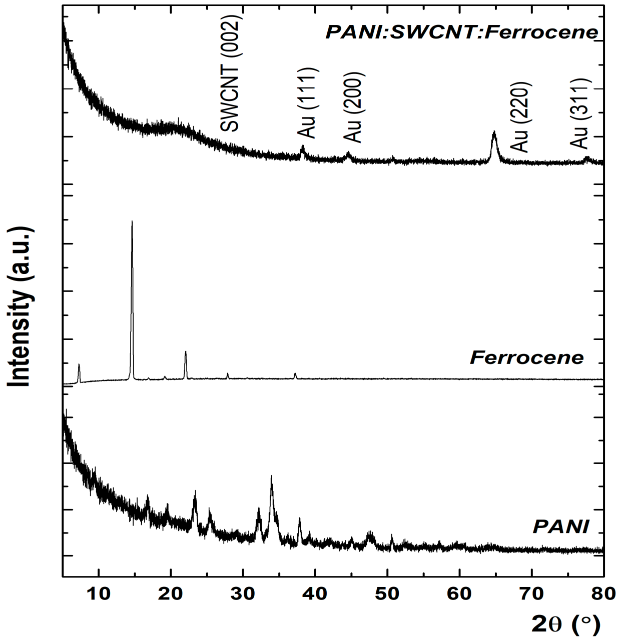


**Figure S6. XRD for PANI, Ferrocene and PANI:PSS:SWCNT:Fc**

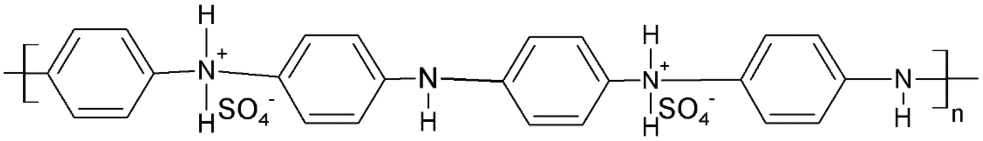

**Figure S7.** Polyaniline as Emeraldine salt, in sulfuric acid media.

**Adsorption-desorption kinetics**

The Langmuir adsorption model for CO gas adsorption-desorption kinetics was used on the new hybrid layer, PANI:PSS/SWCNT/Fc using the method previously reported in [56]. Using the Langmuir adsorption model (1st order Langmuir Adsorption-desorption kinetics) the conductive data of sensor response and recovery are fitted with the first exponential rise and decay curves, respectively. This model considers that only top layer participates in CO adsorption and CO molecules are adsorbed by nitrogen atoms of PANI. The reaction may occur via reaction :

$$CO + e^{-} \xrightleftharpoons{k^{+}/k^{-}} CO^{-}_{(ads)}$$

Firstly, the resistive data of response and recovery curves are converted into conductive data and normalized. The conductive data of response was fitted with exponential rise and the recovery data was fitted with exponential decay. The conductance changes for response and for recovery according to [56} are presented below :

$$\Delta G = \Delta G_{max}\left[1 - exp\left(-\frac{1}{t_{res}}\right)\right] \text{, for response cycle}$$

$$\Delta G = \Delta G_{max} \cdot exp\left(-\frac{1}{t_{rec}}\right)\text{, for recovery cycle}$$

where, $\Delta G$and $\Delta G_{max}$are time dependent change of conductance and the change of maximum conductance, respectively.

By tacking the natural logarithms on both sides, the exponential equation can convert in straight lines and $1/t_{res}$ and $1/t_{rec}$ represent the slopes and $t_{res}$ and $t_{rec}$ can be evaluated at various concentrations of gas (Figure S8 (b) and Figure S8(c)

**Figure S8a.** presents the normalized conductance versus time after 300 ppm CO. The calculated response (Fig S8b) for 300 ppm CO was 33 seconds and for recovery (FigS8c) was 143 seconds. For lower concentrations < 100 ppm, the response time was smaller than 30 s and the and recovery time was smaller than 100s.

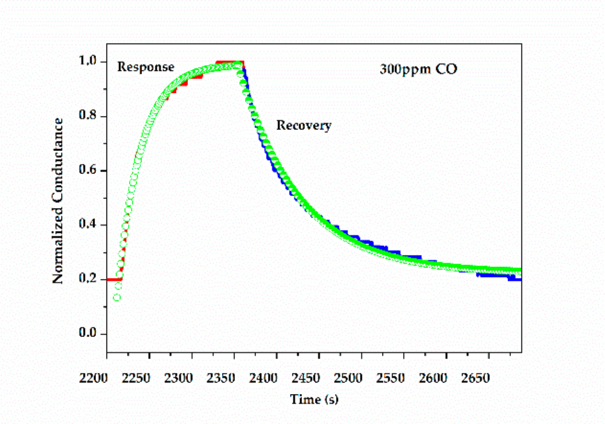


(a)

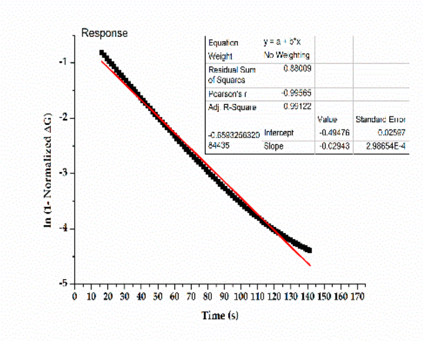


(b)

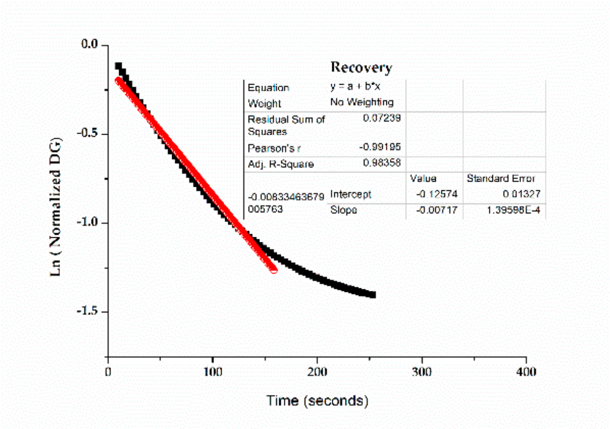


**(c)**

**Figure S8.(a)** Fitted response and recovery curve of PANI:PSS/SWCNT/Fc in presence of 300ppm CO;(b) logharitmic dependence of ln (1-Normalized ΔG) and (c) logharitmic dependence of Normalized ΔG to obtain $1/t_{res}$ and respectivelly $1/t_{rec}$
